# The Marmoset as an Animal Model of Influenza: Infection With A(H1N1)pdm09 and Highly Pathogenic A(H5N1) Viruses via the Conventional or Tracheal Spray Route

**DOI:** 10.3389/fmicb.2018.00844

**Published:** 2018-05-09

**Authors:** Kiyoko Iwatsuki-Horimoto, Noriko Nakajima, Maki Kiso, Kenta Takahashi, Mutsumi Ito, Takashi Inoue, Machiko Horiuchi, Norio Okahara, Erika Sasaki, Hideki Hasegawa, Yoshihiro Kawaoka

**Affiliations:** ^1^Division of Virology, Department of Microbiology and Immunology, Institute of Medical Science, University of Tokyo, Tokyo, Japan; ^2^Department of Pathology, National Institute of Infectious Diseases, Tokyo, Japan; ^3^Marmoset Research Department, Central Institute for Experimental Animals, Kawasaki, Japan; ^4^BioSciences Group, Summit Pharmaceuticals International Corporation, Tokyo, Japan; ^5^Keio Advanced Research Center, Keio University, Tokyo, Japan; ^6^Influenza Research Institute, Department of Pathobiological Sciences, School of Veterinary Medicine, University of Wisconsin-Madison, Madison, WI, United States; ^7^Department of Special Pathogens, International Research Center for Infectious Diseases, Institute of Medical Science, University of Tokyo, Tokyo, Japan

**Keywords:** influenza virus, animal model, marmoset, non-human primate, A(H5N1)

## Abstract

To control infectious diseases in humans, it is important to understand the pathogenicity of the infecting organism(s). Although non-human primates, such as cynomolgus and rhesus macaques, have been used for influenza virus infection models, their size can limit their use in confined animal facilities. In this study, we investigated the susceptibility of marmosets to influenza viruses to assess the possibility of using these animals as a non-human primate model for influenza research. We first used an influenza A (H1N1)pdm09 virus to compare two inoculation routes: the conventional route, via a combination of the intratracheal, intranasal, ocular, and oral routes; and the tracheal spray route. In marmosets inoculated via the tracheal spray route, we found inflammation throughout the lungs and trachea. In contrast, in marmosets inoculated via the conventional route, the inflammation was confined to roughly the center of the lung. These data suggest that the tracheal spray route may be more suitable than the conventional route to inoculate marmosets with influenza viruses. We also tested an influenza A(H5N1) highly pathogenic avian influenza (HPAI) virus and found that some marmosets inoculated with this virus via the tracheal spray route showed weight loss, decreased body temperature, and loss of appetite and activity. The replication of this H5N1 virus in respiratory organs was confirmed. These results indicate the potential of marmosets as an animal model for infection with seasonal or HPAI viruses.

## Introduction

Animal models are essential to the study of infectious diseases in humans. In influenza research, several kinds of animals are used for this purpose (Barnard, 2009; O’Donnell and Subbarao, 2011; Wright et al., 2013). Among them, non-human primates (NHPs) are attractive due to their anatomical remembrance to humans and the availability of a variety of reagents. In fact, rhesus (*Macaca mulatta*) (Chen et al., 2006; Fan et al., 2009; Weinfurter et al., 2011; Shinya et al., 2012), cynomolgus (*Macaca fascicularis*) (Rimmelzwaan et al., 2001, 2003; Kuiken et al., 2003; Baskin et al., 2009; Itoh et al., 2009; Watanabe et al., 2013; Muramoto et al., 2014; Marriott et al., 2016; Shichinohe et al., 2016; Kiso et al., 2017; Nakayama et al., 2017; Wonderlich et al., 2017), and pig-tailed (*Macaca nemestrina*) (Baskin et al., 2004; Baas et al., 2006) macaques have all been used. Although seasonal human influenza viruses or low pathogenic avian influenza A(H7N9) viruses isolated from a patient replicate in the respiratory tract of macaques, these infected animals show mild or no clinical symptoms (Baskin et al., 2004; Watanabe et al., 2013; Shichinohe et al., 2016; Kiso et al., 2017). Even upon infection with highly pathogenic avian influenza (HPAI) A(H5N1) or A(H7N9) viruses, which induce severe lesion in the respiratory tract of macaques, their clinical symptoms range from asymptomatic to mild (Fan et al., 2009; Shinya et al., 2012; Muramoto et al., 2014; Imai et al., 2017; Watanabe et al., 2018) except for some severe (Rimmelzwaan et al., 2001, 2003) or lethal cases (Baskin et al., 2009; Muramoto et al., 2014; Wonderlich et al., 2017). Similarly, although influenza A(H1N1)pdm09 viruses isolated in the early stage of the 2009 pandemic, which caused severe infections in humans and induced severe lung damage, the clinical symptoms of infected macaques ranged from asymptomatic (Itoh et al., 2009; Josset et al., 2012; Watanabe et al., 2012; Marriott et al., 2016) to mild (Safronetz et al., 2011; Mooij et al., 2015). Thus, macaques do not necessarily mimic influenza in humans.

Another issue we need to consider in an animal model of influenza viruses is the inoculation route. Often, NHPs are inoculated with virus via a combination of the intratracheal, intranasal, ocular, and oral routes (i.e., the conventional route) (Itoh et al., 2009; Watanabe et al., 2013; Muramoto et al., 2014). Clearly, delivering a highly concentrated virus-containing liquid to multiple sites in this way is different from an actual infection. Previously, we and others tested the aerosol route for influenza inoculation of cynomolgus macaques, and found it to be more effective than the conventional route (Marriott et al., 2016; Wonderlich et al., 2017; Watanabe et al., 2018). However, it is difficult to deliver an accurate amount of virus via the aerosol route. In contrast, the tracheal spray route can deliver precise liquid aerosol doses to the lung.

Moncla et al. (2013) showed that inoculation of the common marmoset (*Callithrix jacchus*) with A/California/07/2009 A(H1N1)pdm09 led not only to virus replication but also to human-like symptoms such as sneezing, nasal discharge, labored breathing, and lung damage. Similarly, Mooij et al. (2015) showed that common marmosets were susceptible to infection with A/Mexico/InDRE4487/2009 A(H1N1)pdm09. Some of their infected animals experienced labored breathing, loss of appetite, and one animal had persistent fast breathing and displayed tremors; however, none of these animals experienced sneezing, nasal discharge, coughing, or weight loss (Mooij et al., 2015). In this study, we performed a comparative examination of the conventional route and the tracheal spray route of inoculation of marmosets with A(H1N1)pdm09 virus. We also investigated the susceptibility of marmosets to HPAI A(H5N1) virus to assess the possibility of using these animals as a non-human primate model for influenza research.

## Materials and Methods

### Animals

Common Marmosets (*C. jacchus*), free from *Salmonella*, *Shigella*, and *Yersinia*, were bred for this study at the Marmoset Research Department, Central Institute for Experimental Animals (Kawasaki, Japan). Three- to eight-year-old female marmosets (body weight, 295–403 g), were transferred to a biosafety level 3 facility at the University of Tokyo. One week before infection, each marmoset was moved into an isolation cage and placed in a separated isolator for acclimatization. Animals were provided wooden perches and a resting place made of wood as environmental enrichment, and were fed a balanced commercial primate diet (CMS-1M; CLEA Japan) supplemented with honey (Kato bihouen-honpo, Tokyo, Japan) and tap water *ad libitum*. The food was moistened with hot water to vary its texture. In addition to the standard diet, animals were fed sponge cake or marshmallows by animal caretakers. The room was maintained at a constant temperature (27°C) and relative humidity (50%) on a 12:12-h light/dark cycle. Therapeutic treatment with antibiotics and/or analgesics was not provided, because such treatment would have affected the experimental results. The research protocol used is in accordance with the Regulations for Animal Care of the University of Tokyo and the Guidelines for Proper Conduct of Animal Experiments by the Science Council of Japan, and was approved by the Animal Experiment Committee of the Institute of Medical Science, the University of Tokyo (approval number: PA14-30).

### Cells and Viruses

Madin-Darby canine kidney (MDCK) cells were maintained in Eagle’s minimal essential medium (MEM) containing 5% newborn calf serum at 37°C in 5% CO_2_. Human pandemic A(H1N1) virus A/Osaka/164/2009 (Osaka164) (Itoh et al., 2009) and highly pathogenic A(H5N1) virus A/Vietnam/1203/2004 (VN1203) (Muramoto et al., 2014) were propagated in MDCK cells with MEM containing 0.3% bovine serum albumin (BSA).

### Plaque Assay

Viruses were diluted in MEM containing 0.3% BSA. Confluent monolayers of MDCK cells were washed with MEM containing 0.3% BSA, infected with diluted viruses, and incubated for 30–60 min at 37°C. After the virus inoculum was removed, the cells were washed with MEM containing 0.3% BSA and overlaid with a 1:1 mixture of 2× MEM/0.6% BSA and 2% agarose containing 1 μg/ml tosylsulfonyl phenylalanyl chloromethyl ketone (TPCK)-trypsin. Plates were incubated at 37°C for 48 h before virus plaques were counted.

### Experimental Infection of Marmosets With A/Osaka/194/2009 [Osaka164: A(H1N1)pdm09]

Under anesthesia with isoflurane, four animals were inoculated with 1.0 × 10^7^ PFU (in 200 μl) of Osaka164 (pdmH1N1) either via the tracheal spray route with a MicroSprayer^®^ Aerosolizer (liquid) – Model IA-1B (Penn-Century, Inc., Wyndmoor, PA, United States) or via a combination of the intratracheal (100 μl), intranasal (20 μl per nostril), ocular (10 μl per eye), and oral (40 μl) routes. To administer sufficient amounts of virus, we infected the animals with a high dose of virus (1.0 × 10^7^ PFU). We observed the marmosets twice daily, once in the morning and once in the evening, during the experiment to monitor their clinical symptoms. Every other day, the body weight and temperature of each marmoset was measured, nasal aspirates were collected for virus titration, and the animals’ respiratory organs were scanned by means of micro-CT (Rigaku Corporation, Japan) until 15 days post-infection (dpi). Body temperature were measured in the rectum without anesthesia; other treatments were performed under anesthesia with isoflurane.

### Experimental Infection of Marmosets With A/Vietnam/1203/2004 [VN1203: A(H5N1)]

Under anesthesia with isoflurane, seven animals were inoculated with 1.0 × 10^7^ PFU (in 200 μl) of VN1203 A(H5N1) via the tracheal spray route. We observed the marmosets twice daily, once in the morning and once in the evening, during the experiment to monitor their clinical symptoms. Every other day, the body weight and temperature of each marmoset was measured, and nasal aspirates were collected for virus titration. Three or four marmosets were euthanized on 3 or 6 dpi, respectively, and their organs were collected (lungs, bronchus, trachea, nasal turbinate, heart, liver, spleen, kidney, duodenum, rectum, and tonsil), and homogenized with MEM containing 0.3% BSA. Virus titers in these homogenized organs were determined by use of plaque assays in MDCK cells.

### Pathological Examination

Excised tissues were fixed in 4% paraformaldehyde phosphate (PFA) buffer solution for 48 h and processed for paraffin embedding. Nasal samples were immersed in EDTA solution for decalcification after being fixed in PFA. The paraffin blocks were cut into 3-μm-thick sections and were mounted on silane-coated glass slides. To detect sialic acid linked to galactose by an α-2,6 linkage (SAα2,6Gal) or an α-2,3 linkage (SAα2,3Gal), the sections were pre-treated with 0.05% trypsin (DIFCO Laboratories, Detroit, MI, United States) at 37°C for 15 min and with 0.3% hydrogen peroxide at room temperature for 30 min. Then, they were incubated at 4°C, overnight with biotin-conjugated *Sambucus nigra* lectin I (SNA I; EY Laboratories) for SAα2,6Gal detection or biotinylated *Maackia amurensis* Lectin II (MAA II; Vector Laboratories) for SAα2,3Gal detection. After being washed, the sections were incubated with HRP-conjugated streptavidin and were visualized by staining with 3,3′-diaminobenzidine (DAB). The sections were also stained using a standard hematoxylin and eosin procedure and each serial section was processed for immunohistochemistry with a mouse monoclonal antibody for type A influenza nucleoprotein antigen (prepared in our laboratory) that reacts comparably with both of the viruses used in this study. Specific antigen–antibody reactions were visualized with DAB staining by using the DAKO Envision system (DAKO Cytomation).

## Results

### Distribution of Sialic Acids in the Respiratory Tract of a Marmoset

**FIGURE 1:**
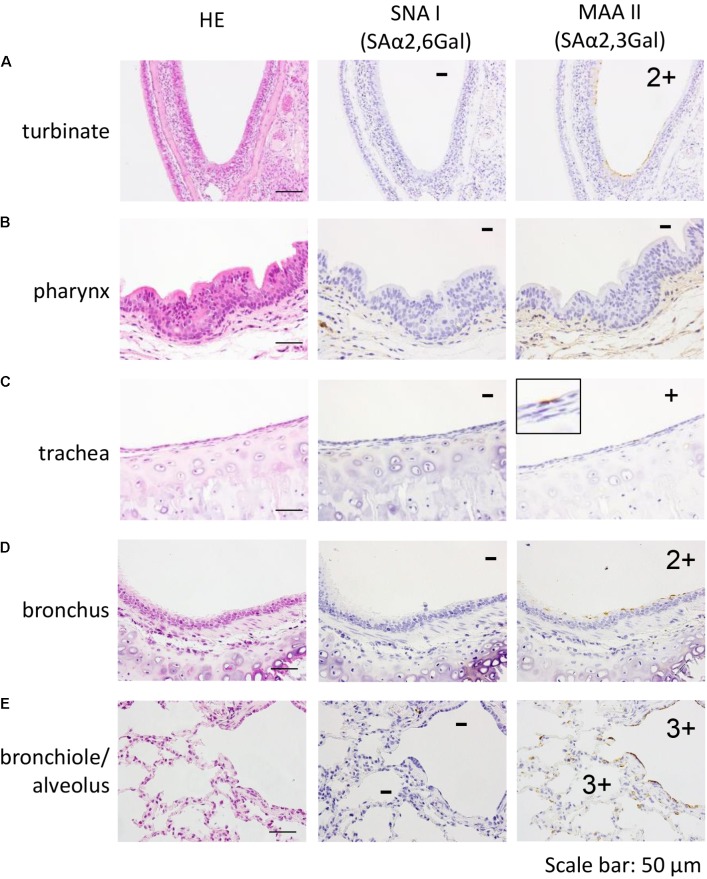
Detection of SAα2,6Gal and SAα2,3Gal oligosaccharides in the nasal turbinate **(A)**, pharynx **(B)**, trachea **(C)**, bronchus **(D)**, and bronchiole/alveolar **(E)** of a 5-year-old marmoset. No section reacted with SNA I (**A–E**, center). In contrast, MAA II reacted with epithelial cells in the nasal turbinate, trachea, bronchus, bronchiole, and alveolus (**A**,**C–E**, right). Quantification of MAA II-positive cells in each section: none (–), a few (+), several and partial (2+), many and diffuse (3+).

First, we examined the sialyloligosaccharide distribution in the respiratory tract of a 5-year-old female marmoset. In the nasal turbinate, pharynx, trachea, bronchus, bronchiole, and alveolus, none of the sections reacted with SNA I, which is specific for SAα2,6Gal (**Figures 1A–E**, center). In contrast, epithelial cells that bind SAα2,3Gal-specific MAA II were detected in the nasal turbinate, trachea, bronchus, bronchiole, and alveolus (**Figures 1A,C–E**, right).

### Comparison of Influenza Virus Inoculation Routes

**FIGURE 2:**
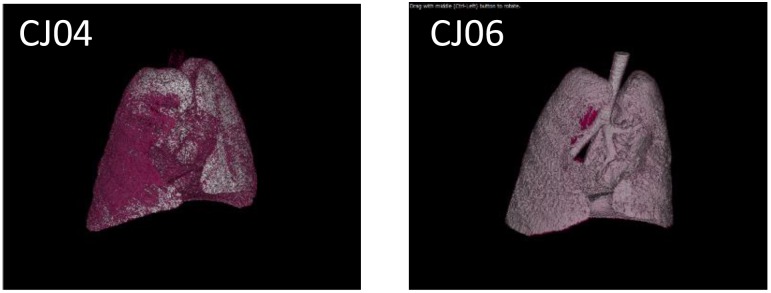
Micro-CT of VN1203-infected marmosets. CJ04 was infected via the tracheal spray route; CJ06 was infected via the conventional route. Images of the lungs of marmosets CJ04 and CJ06 on 3 dpi. The pink color indicates inflamed areas.

We next performed a pilot study using a limited number of animals to determine a suitable inoculation route by comparing inoculation with Osaka164 A(H1N1)pdm09 virus (1.0 × 10^7^ PFU/200 μl/animal) via two routes: the tracheal spray route (200 μl), and the conventional route, which is a combination of the intratracheal (100 μl), intranasal (20 μl per nostril), ocular (10 μl per eye), and oral (40 μl) routes. Four 4- to 8-year-old female marmosets were intramuscularly anesthetized and intratracheally inoculated via the tracheal spray route (*n* = 2, animal IDs: CJ03 and CJ04) or via the conventional route (*n* = 2, animal IDs: CJ05 and CJ06). None of the infected marmosets showed any clinical signs or changes in body weight or temperature (data not shown). One of the animals inoculated via the tracheal spray route and both animals inoculated via the conventional route shed virus from their nasal cavity until 7 or 11 dpi, respectively, (**Table 1**). We also scanned the whole lungs of the animals every other day by using micro-CT. Inflammation in CJ04 was spread throughout the lung and trachea on 3 dpi (**Figure 2**, left), whereas inflammation in CJ06 was confined to around the center of the lung (**Figure 2**, right). These data suggest that the tracheal spray can evenly deliver virus to a wider area of lungs than the conventional method.

**Table 1:** Virus titers in the nasal aspirate of marmosets infected with Osaka164^a^.

Inoculation route	Animal ID	Virus titers (log_10_PFU/ml)						
		
		Days post-inoculation						
		
		1	3	5	7	9	11	13
Tracheal spray route	CJ03	–	–	–	–	–	–	–
	CJ04	1.4	4.7	–	3.2	–	–	–
Conventional route	CJ05	1.0	1.8	2.1	–	5.2	1.3	–
	CJ06	1.6	3.7	2.8	2.4	–	–	–


*^*a*^Four- to eight-year-old female marmosets were anesthetized and inoculated with 1.0 × 10^*7*^ PFU (in 200 μl) of Osaka164 via the tracheal spray route (CJ03 and CJ04) or via the conventional route (CJ05 and CJ06). Under anesthesia, nasal aspirates from each marmoset were collected every other day for virus titration.*

### Growth Properties of A(H5N1) Virus in Marmosets

**FIGURE 3:**
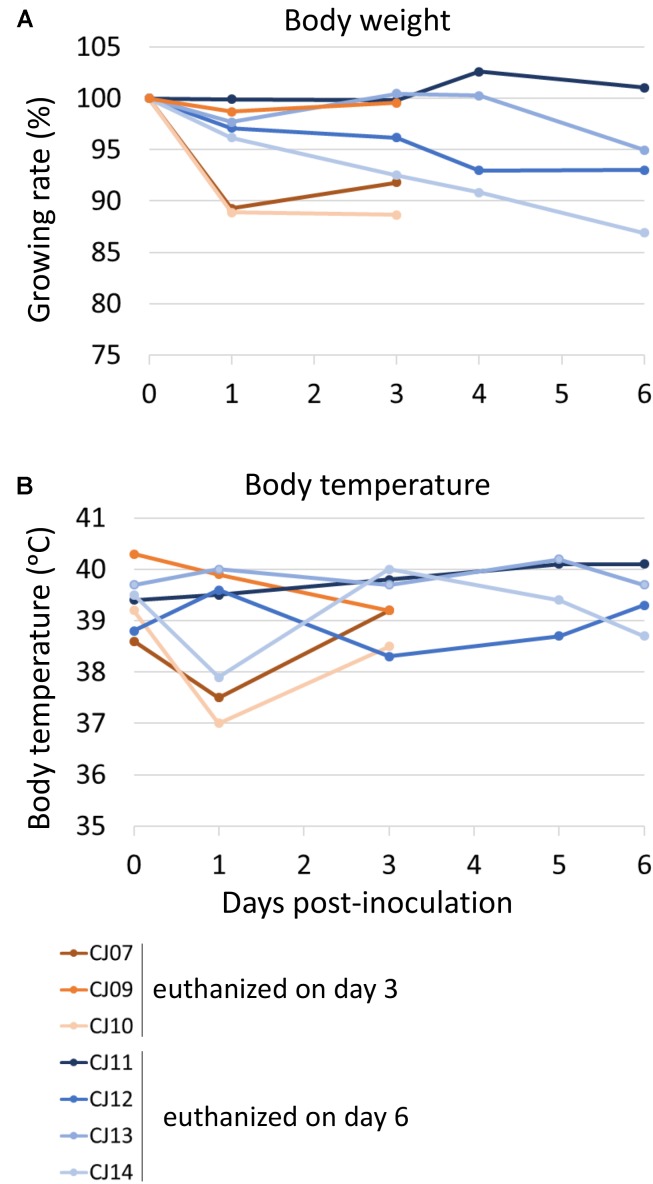
Change in body weight **(A)** and body temperature **(B)** of VN1203-infected marmosets. On 0 (the day of infection), 1, 3, 5, and 6 dpi, the body weight **(A)** and temperature **(B)** of each marmoset was measured. Three (CJ07, CJ09, and CJ10) or four (CJ11, CJ12, CJ13, and CJ14) marmosets were euthanized on 3 or 6 dpi, respectively.

To evaluate the suitability of the marmoset model for the study of HPAI virus infection, 3- to 7-year-old female marmosets were anesthetized and inoculated with 1.0 × 10^7^ PFU of VN1203 A(H5N1) virus (*n* = 7, animal IDs CJ07, CJ09–14) via the tracheal spray route. Two animals (CJ07 and CJ10) showed sudden weight loss after 1 dpi, and two other animals (CJ12 and CJ14) showed moderate weight loss during the observation period (**Figure 3A**). Three animals on 1 dpi (CJ07, CJ10, and CJ14) and one animal on 3 dpi (CJ12) showed decreased body temperature, but the temperature of all four animals returned to normal on the next measuring date (**Figure 3B**). CJ07 and CJ14 showed loss of appetite and reduced activity from 2 dpi to the final day of the observation period, but they did not cough or sneeze (data not shown). On 3 (*n* = 3; CJ07, CJ09, and CJ10) and 6 (*n* = 4; CJ11, CJ12, CJ13, and CJ14) dpi, animals were euthanized and their organs were collected for virological and pathological examination. High titers of VN1203 were detected in all of the respiratory organs and other internal organs on 3 dpi although at this time point, the virus was recovered from only the cerebellum of one animal (**Table 2**). By 6 dpi, the virus titers had declined in many organs; however, the virus was recovered from all parts of the brain of one animal (CJ14). Although there was some variability among the virus titers, as is commonly observed among outbred animals, these results indicate that marmosets are susceptible to A(H5N1) virus.

**Table 2:** Virus titers in organs of infected marmosets^a^.

Organs		Virus titers (log_10_PFU/g)						
		
		3 dpi			6 dpi			
			
		CJ07	CJ09	CJ10	CJ11	CJ12	CJ13	CJ14
Left lung	Cranial	7.2	3.3	6.7	–^b^	–	3.2	–


	Caudal	6.2	3.9	6.6	–	–	2.9	–


Right lung	Cranial	6.7	4.1	6.8	–	3.7	3.9	–


	Middle	7.6	5.5	5.6	–	2.7	2.7	–


	Caudal	8.0	4.0	6.2	–	4.3	2.7	–


	Accessary	7.3	3.7	6.5	–	–	–	–


Trachea		5.6	3.4	4.7	–	6.0	2.4	3.0


Left bronchus		7.7	3.2	6.9	–	3.3	5.0	3.4


Nasal turbinate		6.1	5.7	4.6	5.3	–	6.5	4.4


Cerebrum	Frontal	–	–	–	–	–	–	3.0


	Temporal	–	–	–	–	–	–	3.2


	Parietal	–	–	–	–	–	–	3.3


	Occipital	–	–	–	–	–	–	4.4


Brain stem		–	–	–	–	–	–	6.8


Olfactory bulb		–	–	–	–	–	–	4.9


Cerebellum		4.5	–	–	–	–	3.7	5.1


Heart		4.2	3.4	3.6	–	–	–	–


Liver		4.7	–	3.2	–	–	–	–


Duodenum		4.6	4.5	4.0	–	–	–	–


Colon		4.2	–	6.0	–	–	–	2.0


Spleen		3.8	–	4.1	–	–	2.7	–


Kidney		4.2	–	2.2	–	–	–	–


Serum		–	–	–	–	–	–	–


Whole blood		ND^c^	ND	ND	–	–	–	–




*^*a*^Marmosets were inoculated with 1.0 × 10^*7*^ PFU (in 200 μl) of VN1203 A(H5N1) via the tracheal spray route; ^*b*^–, virus not detected (detection limit: 1.0 log_*10*_PFU/ml); ^*c*^ND, not done.*

### Pathological Analyses of A(H5N1) Virus-Infected Marmosets

**FIGURE 4:**
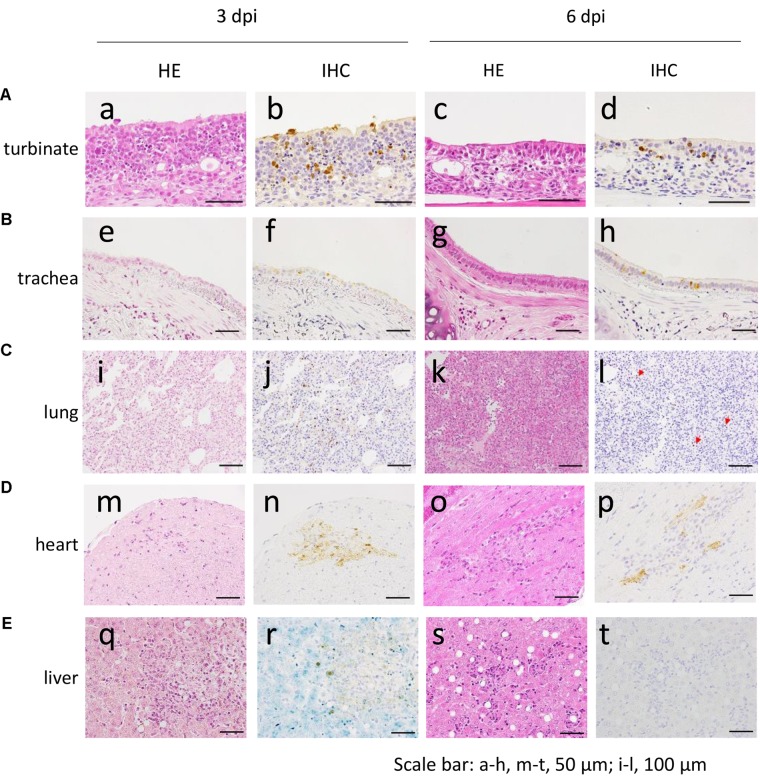
Pathological examination of the nasal turbinate **(A)**, trachea **(B)**, lung **(C)**, heart **(D)**, and liver **(E)** of VN1203-infected marmosets. a, b, m, n, CJ09; c, d, s, t, CJ11; e, f, i, j, q, r, CJ07; g, h, CJ14; k, l, CJ13; o, p, CJ12. Red arrows in (l) indicate antigen-positive cells. HE, hematoxylin and eosin staining; IHC, immunohistochemistry for the detection of influenza virus NP antigen.

**FIGURE 5:**
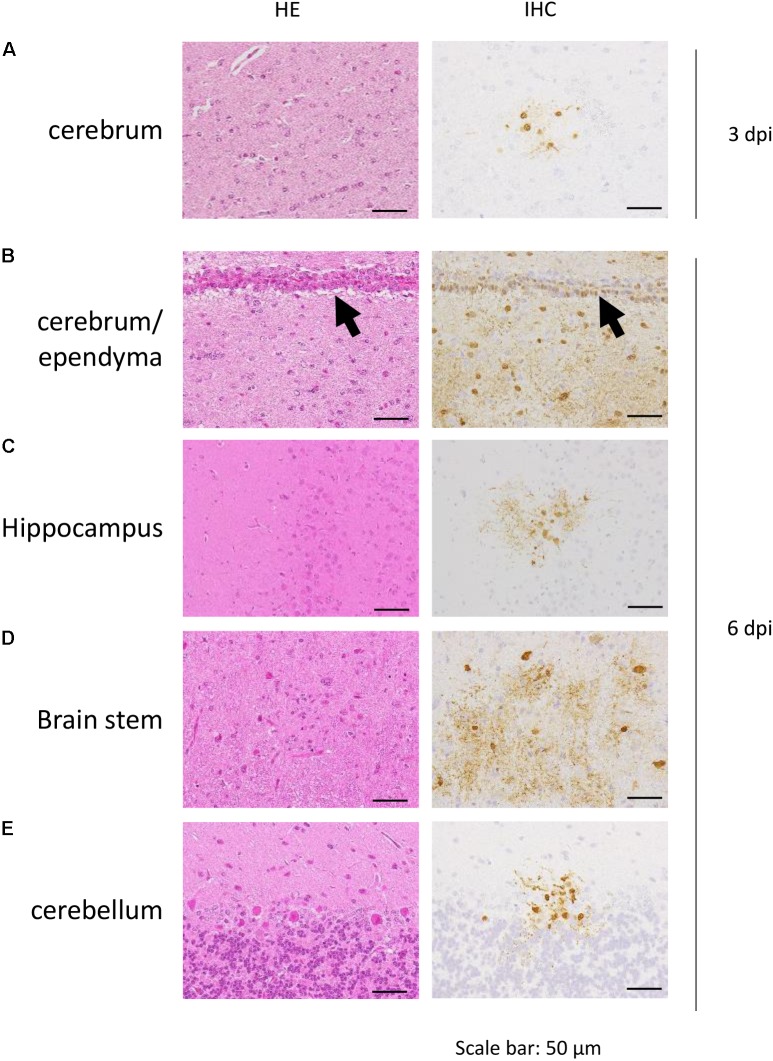
Pathological examination of marmoset brain. Cerebrum of CJ07 **(A)**, cerebrum of CJ14 **(B)**, hippocampus of CJ14 **(C)**, brain stem of CJ13 **(D)**, and cerebellum of CJ13 **(E)**, which were infected with VN1203. Black arrows in **(B)** indicate ependyma. HE, hematoxylin and eosin staining; IHC, immunohistochemistry for the detection of influenza virus NP antigen.

The distribution of antigen-positive cells detected by immunohistochemistry (**Tables 3A,B**) showed almost the same pattern as that of the virus titers (**Table 2**). Viral antigen was detected in the nasal turbinate and trachea in both the 3 and 6 dpi groups (**Figures 4A,B** and **Table 3A**). All lung sections showed inflammation, and the viral antigen-positive signal was more widely observed in CJ07 and CJ10 than the other animals (**Figure 4C** and **Table 3A**). Some of the heart and liver samples showed focal inflammation as well as viral antigen-positive cells (**Figures 4D,E** and **Table 3B**). In the brain, only a few sections showed mild focal inflammation; however, immunohistochemistry revealed some neural cells that were positive for viral antigen, particularly many ependymal cells in CJ14 (**Figure 5** and **Table 3B**). These findings indicate that VN1203 infected and replicated not only in the respiratory organs but also in the heart, liver, and brain of marmosets.

**Table 3A:** Pathologic scores^a,b^ of respiratory organs by hematoxylin and eosin staining (HE) and the number of antigen-positive cells^c^ by immunohistochemistry (IHC) in VN1203-infected marmosets.

Organs		3 dpi						6 dpi							
			
		CJ07		CJ09		CJ10		CJ11		CJ12		CJ13		CJ14	
								
		HE	IHC	HE	IHC	HE	IHC	HE	IHC	HE	IHC	HE	IHC	HE	IHC
Left lung	Cranial	4	+	3	-	5	+	4	-^b^	4	-	5	-	3	-


	Caudal	4	+	3	-	5	+	3	-	4	-	5	-	4	-


Right lung	Cranial	4	+	3	-	5	+	3	-	5	+	4	+	5	-


	Middle	4	+	3	-	4	+	3	-	5	-	5	+	4	-


	Caudal	3	+	3	-	5	+	3	-	5	-	5	-	5	-


	Accessary	NA^d^	NA	NA	NA	NA	NA	NA	NA	NA	NA	NA	NA	NA	NA


Trachea		1	+	1	-	1	-	1	-	1	+	1	-	1	+


Nasal turbinate		1	+	1	+	2	+	1	+	1	-	1	+	1	+




*^*a*^Lung: -, no apparent changes; 1, minimal changes; 2, slight inflammation; 3, mild inflammation including infiltration of neutrophils, monocytes/macrophages, or lymphocytes; 4, moderate inflammation (>50% of sections examined); 5, marked inflammation including edema and fibrin transudation. The area with edema and fibrin transudation represents <50% of the section examined; 6, severe inflammation. The area with edema and fibrin transudation represents >50% of the section examined.^*b*^Trachea and nasal turbinate: -, no apparent changes; 1, mild inflammation; 2, moderate inflammation; 3, severe inflammation. ^*c*^Detection of influenza virus antigen in tissue sample sections. The extent of the abundance of virus-antigen-positive cells in the entire section of the sample was defined as follows: -, no antigen-positive cells found; +, 1–10 virus-antigen-positive cells found; ++, 11–101 virus-antigen-positive cells found; +++, > 101 virus-antigen-positive cells found. ^*d*^NA, not available.*

**Table 3B:** Pathologic scores^a^ of non-respiratory organs by hematoxylin and eosin staining (HE) and the number of antigen-positive cells^b^ by immunohistochemistry (IHC) in VN1203-infected marmosets.

Organs		3 dpi						6 dpi							
			
		CJ07		CJ09		CJ10		CJ11		CJ12		CJ13		CJ14	
								
		HE	IHC	HE	IHC	HE	IHC	HE	IHC	HE	IHC	HE	IHC	HE	IHC
Cerebrum	Frontal	-	-	-	-	-	-	-	-	-	-	-	-	-	+++
	Temporal	-	-	-	-	-	-	-	-	-	+	-	+	-	+++
	Parietal	-	+	-	-	1	-	-	-	-	-	-	-	-	+++
	Occipital	-	+	-	-	-	-	-	-	-	-	-	-	-	++
Brain stem		-	-	-	-	-	-	-	-	1	+	1	+	-	+++
Olfactory bulb		-	-	-	-	NA^c^	NA	-	-	-	-	-	-	-	-
Cerebellum		-	+	-	-	-	-	-	-	-	-	-	+	-	-
Heart		-	++	1	++	-	+	-	-	1	+	-	+	-	NA
Liver		1	++	-	-	1	+	1	-	-	-	-	-	-	-
Duodenum		-	-	-	-	-	-	-	-	-	-	NA	NA	NA	NA
Colon		-	-	-	-	-	-	-	-	-	-	-	-	-	-
Spleen		-	-	-	-	-	-	-	-	-	-	-	-	-	NA
Kidney		-	-	-	-	-	-	-	-	-	-	-	-	-	-


*^*a*^-, No apparent changes; 1, mild inflammation; 2, moderate inflammation; 3, severe inflammation. ^*b*^Detection of influenza virus antigen in tissue sample sections. The extent of the abundance of virus-antigen-positive cells in the entire section of the sample was defined as follows: -, no antigen-positive cells found; +, 1–10 virus-antigen-positive cells found; ++, 11–101 virus-antigen-positive cells found; +++, > 101 virus-antigen-positive cells found. ^c^NA, not available.*

## Discussion

Moncla et al. (2013) showed that marmosets infected with A/California/07/2009 A(H1N1)pdm09 virus via the conventional route (a combination of intratracheal, oral, intranasal, and ocular inoculation) supported replication of the virus, and exhibited symptoms commonly seen in humans, including sneezing, nasal discharge, and labored breathing (Moncla et al., 2013). Mooij et al. (2015) similarly found that marmosets infected with A/Mexico/InDRE4487/2009 A(H1N1)pdm09 virus via the intratracheal route (using a catheter inserted into the trachea) supported replication of the virus, and displayed labored breathing and loss of appetite but did not experience sneezing, nasal discharge, or coughing. In contrast, in our study, we found that a different A(H1N1)pdm09 strain (Osaka164) did not cause any symptoms in marmosets infected via either the conventional or intratracheal spray route although this virus caused clear respiratory symptoms (e.g., sneezing) in ferrets (Kiso et al., 2010a). This difference in tissue tropism likely reflects the difference in the virus strains used. Although we could not reproduce the previous finding of clinical symptoms in this animal species with an A(H1N1)pdm09 strain, our findings indicate that marmosets are highly sensitive to this virus.

VN1203 A(H5N1)-infected marmosets showed symptoms such as loss of appetite and reduced activity but not respiratory symptoms such as sneezing, nasal discharge, or labored breathing. However, virus replication was observed not only in the respiratory tract of the marmosets but also other organs including brain and heart (**Figures 4**, **5** and **Tables 2**, **3B**). Previous studies of A(H5N1)-infected patients have detected A(H5N1) viruses outside the respiratory tract in brains, intestines, spleen, and circulating mononuclear cells (Uiprasertkul et al., 2005; Gu et al., 2007). Rimmelzwaan et al. (2001) found that A/Hong Kong/156/97 (H5N1) replicated not only in the respiratory tract but also in the heart of cynomolgus macaques. In our previous study of cynomolgus macaques infected with A/Vietnam/UT3040/2004 [H5N1; note that A/Vietnam/UT3040/2004(H5N1) and VN1203 were isolated from the same patient and their pathogenicity in animal models is similar (Kiso et al., 2010b)], we readily detected virus in the respiratory organs and conjunctiva, but not in other systemic organs, (Muramoto et al., 2014), and we detected virus in only a portion of the brain (only in the occipital lobe of 1 of 6 animals), in the duodenum (1 of 6 animals), and in the mediastinal lymph node (3 of 6 animals) (Watanabe et al., 2018). In this study, VN1203 A(H5N1) virus replicated in various organs of marmoset (**Figures 4**, **5** and **Tables 2**, **3B**). This result suggests that marmosets are more susceptible to VN1203 A(H5N1) than are cynomolgus macaques.

In this study, we tested the tracheal spray route as a novel infection route. Previously, we found that the conventional infection route results in rather localized replication of influenza viruses in the lungs of rhesus (Shinya et al., 2012) and cynomolgus macaques (Itoh et al., 2009; Watanabe et al., 2013; Muramoto et al., 2014; Kiso et al., 2017). Similarly, in the present study, inoculation via the conventional route led to a strong local infection (**Figure 2**). In contrast, inoculation via the tracheal spray route led to virus replication throughout the lung (**Figure 2**). A(H5N1) infection via the tracheal spray route produced only limited variations in the pathologic scores for the lung lobes of each animal (**Table 3A**). These results suggest that the tracheal spray route better represented an actual infection than did the conventional route. In this study, 1 of 2 (50%) animals infected via the tracheal spray route did not shed virus, whereas 2 of 2 (100%) animals inoculated via the conventional route shed virus (**Table 1**). Similarly, in our cynomolgus macaque experiments, we found that 1 of 4 (25%) of the animals infected via the aerosol route did not shed virus, whereas 3 of 3 (100%) animals infected via the conventional route shed virus (Watanabe et al., 2018). Since virus is directly inoculated into the nostril by the conventional route, it would be easier for the virus to replicate and be detected at the inoculation site by nasal swabbing than when the aerosol or tracheal spray route is used. Virus replication in CJ03 (**Table 1**) and CJ11 (**Table 2**), which were infected via the tracheal spray route, was limited, which may have been due to technical issues with the virus administration. Alternatively, these animals may have been immune to influenza viruses, although we screened the animals to ensure that they lacked neutralizing antibodies against influenza viruses before we infected them. Some variability in virus titers among individual animals has been reported previously with outbred animal models (Rimmelzwaan et al., 2001; Muramoto et al., 2014; Watanabe et al., 2018). Although further adjustment may be needed to optimize the tracheal spray route as a method of inoculation (e.g., determining the optimal depth of insertion of the spray, inoculum volume, etc.), this route appears to be suitable as an alternative approach to influenza virus inoculation.

By using SNAI and MAAII lectin staining, we found that only SAα2,3Gal was detectable in the respiratory tract of the marmoset; SAα2,6Gal was not detected (**Figure 1**). Yet, high replication of VN1203 A(H5N1) human virus, which preferentially binds to SAα2,6Gal over SAα2,3Gal, was observed in respiratory tissues (**Figures 4**, **5** and **Tables 2**, **3**). These findings may suggest that the SAα2,6Gal that is present in the respiratory tract of marmoset is not detectable with SNAI. Further studies are needed to test this possibility.

One limitation of our study, and all NHP studies, is that the number of animals used must be small for ethical reasons. Despite these small numbers, however, valuable information can be obtained; for example, extensive immunological studies are possible due to the availability of abundant immunological reagents. In conclusion, our data show that marmosets can be used in influenza research for pathogenicity studies and also live imaging by micro-CT. Marmosets have potential as a primate model for studies of human and HPAI virus infection.

## Author Contributions

YK conceived and designed the experiments. KI-H, NN, MK, KT, MI, TI, MH, and NO conducted the experiments. ES, HH, and YK contributed reagents, materials, and analysis tools. KI-H, NN, and YK wrote the manuscript. All authors reviewed the manuscript.

## Conflict of Interest Statement

MH is employed by Summit Pharmaceuticals International Corporation. The other authors declare that the research was conducted in the absence of any commercial or financial relationships that could be construed as a potential conflict of interest.

## References

[B1] Baas T. Baskin C. R. Diamond D. L. Garcia-Sastre A. Bielefeldt-Ohmann H. Tumpey T. M. (2006). Integrated molecular signature of disease: analysis of influenza virus-infected macaques through functional genomics and proteomics. 80 10813–10828. 10.1128/jvi.00851-06 16928763 PMC1641753

[B2] Barnard D. L. (2009). Animal models for the study of influenza pathogenesis and therapy. 82 A110–A122. 10.1016/j.antiviral.2008.12.014 19176218 PMC2700745

[B3] Baskin C. R. Bielefeldt-Ohmann H. Tumpey T. M. Sabourin P. J. Long J. P. Garcia-Sastre A. (2009). Early and sustained innate immune response defines pathology and death in nonhuman primates infected by highly pathogenic influenza virus. 106 3455–3460. 19218453 10.1073/pnas.0813234106 PMC2642661

[B4] Baskin C. R. Garcia-Sastre A. Tumpey T. M. Bielefeldt-Ohmann H. Carter V. S. Nistal-Villan E. (2004). Integration of clinical data, pathology, and cDNA microarrays in influenza virus-infected pigtailed macaques (*Macaca nemestrina*). 78 10420–10432. 10.1128/jvi.78.19.10420-10432.2004 15367608 PMC516400

[B5] Chen H. Li Y. Li Z. Shi J. Shinya K. Deng G. (2006). Properties and dissemination of H5N1 viruses isolated during an influenza outbreak in migratory waterfowl in western China. 80 5976–5983. 10.1128/jvi.00110-06 16731936 PMC1472608

[B6] Fan S. Gao Y. Shinya K. Li C. K. Li Y. Shi J. (2009). Immunogenicity and protective efficacy of a live attenuated H5N1 vaccine in nonhuman primates. 5:e1000409. 10.1371/journal.ppat.1000409 19412338 PMC2669169

[B7] Gu J. Xie Z. Gao Z. Liu J. Korteweg C. Ye J. (2007). H5N1 infection of the respiratory tract and beyond: a molecular pathology study. 370 1137–1145. 10.1016/s0140-6736(07)61515-3 17905166 PMC7159293

[B8] Imai M. Watanabe T. Kiso M. Nakajima N. Yamayoshi S. Iwatsuki-Horimoto K. (2017). A highly pathogenic avian H7N9 influenza virus isolated from a human is lethal in some ferrets infected via respiratory droplets. 22 615.e8–626.e8. 10.1016/j.chom.2017.09.008 29056430 PMC5721358

[B9] Itoh Y. Shinya K. Kiso M. Watanabe T. Sakoda Y. Hatta M. (2009). In vitro and in vivo characterization of new swine-origin H1N1 influenza viruses. 460 1021–1025. 10.1038/nature08260 19672242 PMC2748827

[B10] Josset L. Engelmann F. Haberthur K. Kelly S. Park B. Kawoaka Y. (2012). Increased viral loads and exacerbated innate host responses in aged macaques infected with the 2009 pandemic H1N1 influenza A virus. 86 11115–11127. 10.1128/jvi.01571-12 22855494 PMC3457171

[B11] Kiso M. Iwatsuki-Horimoto K. Yamayoshi S. Uraki R. Ito M. Nakajima N. (2017). Emergence of oseltamivir-resistant h7n9 influenza viruses in immunosuppressed cynomolgus macaques. 216 582–593. 10.1093/infdis/jix296 28931216

[B12] Kiso M. Shinya K. Shimojima M. Takano R. Takahashi K. Katsura H. (2010a). Characterization of oseltamivir-resistant 2009 H1N1 pandemic influenza A viruses. 6:e1001079. 10.1371/journal.ppat.1001079 20865125 PMC2928817

[B13] Kiso M. Takahashi K. Sakai-Tagawa Y. Shinya K. Sakabe S. Le Q. M. (2010b). T-705 (favipiravir) activity against lethal H5N1 influenza A viruses. 107 882–887. 10.1073/pnas.0909603107 20080770 PMC2818889

[B14] Kuiken T. Rimmelzwaan G. F. Van Amerongen G. Osterhaus A. D. (2003). Pathology of human influenza A (H5N1) virus infection in cynomolgus macaques (*Macaca fascicularis*). 40 304–310. 10.1354/vp.40-3-304 12724572

[B15] Marriott A. C. Dennis M. Kane J. A. Gooch K. E. Hatch G. Sharpe S. (2016). Influenza A virus challenge models in cynomolgus macaques using the authentic inhaled aerosol and intra-nasal routes of infection. 11:e0157887. 10.1371/journal.pone.0157887 27311020 PMC4911124

[B16] Moncla L. H. Ross T. M. Dinis J. M. Weinfurter J. T. Mortimer T. D. Schultz-Darken N. (2013). A novel nonhuman primate model for influenza transmission. 8:e78750. 10.1371/journal.pone.0078750 24244352 PMC3828296

[B17] Mooij P. Koopman G. Mortier D. van Heteren M. Oostermeijer H. Fagrouch Z. (2015). Pandemic swine-origin H1N1 influenza virus replicates to higher levels and induces more fever and acute inflammatory cytokines in cynomolgus versus rhesus monkeys and can replicate in common marmosets. 10:e0126132. 10.1371/journal.pone.0126132 25946071 PMC4422689

[B18] Muramoto Y. Shoemaker J. E. Le M. Q. Itoh Y. Tamura D. Sakai-Tagawa Y. (2014). Disease severity is associated with differential gene expression at the early and late phases of infection in nonhuman primates infected with different H5N1 highly pathogenic avian influenza viruses. 88 8981–8997. 10.1128/jvi.00907-14 24899188 PMC4136255

[B19] Nakayama M. Itoh Y. Shichinohe S. Nakabayashi R. Ishigaki H. Sakoda Y. (2017). Potential risk of repeated nasal vaccination that induces allergic reaction with mucosal IgE and airway eosinophilic infiltration in cynomolgus macaques infected with H5N1 highly pathogenic avian influenza virus. 35 1008–1017. 10.1016/j.vaccine.2017.01.008 28109707

[B20] O’Donnell C. D. Subbarao K. (2011). The contribution of animal models to the understanding of the host range and virulence of influenza A viruses. 13 502–515. 10.1016/j.micinf.2011.01.014 21276869 PMC3071864

[B21] Rimmelzwaan G. F. Kuiken T. van Amerongen G. Bestebroer T. M. Fouchier R. A. Osterhaus A. D. (2001). Pathogenesis of influenza A (H5N1) virus infection in a primate model. 75 6687–6691. 10.1128/jvi.75.14.6687-6691.2001 11413336 PMC114392

[B22] Rimmelzwaan G. F. Kuiken T. van Amerongen G. Bestebroer T. M. Fouchier R. A. Osterhaus A. D. (2003). A primate model to study the pathogenesis of influenza A (H5N1) virus infection. 47(Suppl.), 931–933. 10.1637/0005-2086-47.s3.931 14575089

[B23] Safronetz D. Rockx B. Feldmann F. Belisle S. E. Palermo R. E. Brining D. (2011). Pandemic swine-origin H1N1 influenza A virus isolates show heterogeneous virulence in macaques. 85 1214–1223. 10.1128/jvi.01848-10 21084481 PMC3020514

[B24] Shichinohe S. Itoh Y. Nakayama M. Ozaki H. Soda K. Ishigaki H. (2016). Comparison of pathogenicities of H7 avian influenza viruses via intranasal and conjunctival inoculation in cynomolgus macaques. 493 31–38. 10.1016/j.virol.2016.03.007 26994587

[B25] Shinya K. Gao Y. Cilloniz C. Suzuki Y. Fujie M. Deng G. (2012). Integrated clinical, pathologic, virologic, and transcriptomic analysis of H5N1 influenza virus-induced viral pneumonia in the rhesus macaque. 86 6055–6066. 10.1128/jvi.00365-12 22491448 PMC3372212

[B26] Uiprasertkul M. Puthavathana P. Sangsiriwut K. Pooruk P. Srisook K. Peiris M. (2005). Influenza A H5N1 replication sites in humans. 11 1036–1041. 10.3201/eid1107.041313 16022777 PMC3371815

[B27] Watanabe T. Imai M. Watanabe S. Shinya K. Hatta M. Li C. (2012). Characterization in vitro and in vivo of pandemic (H1N1) 2009 influenza viruses isolated from patients. 86 9361–9368. 10.1128/JVI.01214-12 22718834 PMC3416174

[B28] Watanabe T. Iwatsuki-Horimoto K. Kiso M. Nakajima N. Takahashi K. Ito M. (2018). Experimental infection of cynomolgus macaques with highly pathogenic H5N1 influenza virus through the aerosol route. 19:4801. 10.1038/s41598-018-23022-0 29556081 PMC5859186

[B29] Watanabe T. Kiso M. Fukuyama S. Nakajima N. Imai M. Yamada S. (2013). Characterization of H7N9 influenza A viruses isolated from humans. 501 551–555. 10.1038/nature12392 23842494 PMC3891892

[B30] Weinfurter J. T. Brunner K. Capuano S. V. III Li C. Broman K. W. Kawaoka Y. (2011). Cross-reactive T cells are involved in rapid clearance of 2009 pandemic H1N1 influenza virus in nonhuman primates. 7:e1002381. 10.1371/journal.ppat.1002381 22102819 PMC3213121

[B31] Wonderlich E. R. Swan Z. D. Bissel S. J. Hartman A. L. Carney J. P. O’Malley K. J. (2017). Widespread virus replication in alveoli drives acute respiratory distress syndrome in aerosolized H5N1 influenza infection of macaques. 198 1616–1626. 10.4049/jimmunol.1601770 28062701 PMC5751439

[B32] Wright P. F. Neumann G. Kawaoka Y. (2013). “Orthomyxoviruses,” in , ed. Knipe H. P. (Philadelphia, PA: Lippincott Williams & Wilkins), 1186–1243.

